# Village-Scale Livelihood Change and the Response of Rural Settlement Land Use: Sihe Village of Tongwei County in Mid-Gansu Loess Hilly Region as an Example

**DOI:** 10.3390/ijerph15091801

**Published:** 2018-08-21

**Authors:** Libang Ma, Shichun Liu, Yiwen Niu, Meimei Chen

**Affiliations:** College of Geography and Environmental Science, Northwest Normal University, Lanzhou 730000, China; liusc0812@163.com (S.L.); niuyw0204@163.com (Y.N.); 18894492718@163.com (M.C.)

**Keywords:** rural household livelihoods, livelihood assets, livelihood change, rural settlement land use, mid-Gansu loess hilly region

## Abstract

Rural livelihood change has great influence on the scale, structure, and morphology of rural settlement land use, thus bringing new challenges to rural revitalization and settlement reconstruction. Sihe village of Tongwei County in mid-Gansu loess hilly region (China) was taken as an example here. Based on participatory rural appraisal data, we analyzed the structure and allocation of rural households’ livelihood assets as well as their livelihood diversity by using ecological asset, livelihood diversification index, and landscape pattern index models. We aimed to find a response mechanism between rural livelihood change and rural settlement land use change. The results might provide useful information for the selection of new village sites, reconstruction of rural settlements, and creation of livable rural environment. Results indicate that: (1) The total value of the average livelihood assets per household in the Sihe village increased significantly from 0.48 in 1988 to 1.288 in 2016. The four types of livelihood assets including natural, material, manpower, and financial assets changed with time. In 1988, the manpower asset was the most important type of livelihood assets, with value accounting for 76.67% of the total value of livelihood assets. With the extension of time, the proportions of the four types of assets in total livelihood assets became closer to each other. The livelihood diversification index of the Sihe village increased from 2.01 in 1988 to 3 in 2016, indicating the rural livelihoods became diverse; (2) Because of the dual influence of external environmental factors and the rural development policies of the country and the region, the livelihoods changed towards agricultural sector from 1988 to 2008, and the agricultural livelihoods tended to be diverse. The following trend of livelihood strategy change was observed: from diverse non-agricultural production group (IV) to agricultural and non-agricultural production group (III), then to diverse agricultural production group (II) and finally to agricultural production group (I). After 2008, the livelihoods changed towards non-agricultural sector, and the non-agricultural livelihoods tended to be diverse. This trend of livelihood change is opposite to that before 2008; (3) 2008 is the key year of livelihood change. Livelihood change caused changes in the scale, structure, and morphology of rural settlement land use, which eventually led to the change of rural residential land use.

## 1. Introduction

The steady progression of new industrialization, healthy urbanization, and agricultural industrialization speeds up the differentiation of rural households in China. Accurately identifying the characteristics and trend of household differentiation in this background can provide a basis for identifying the livelihood challenges that rural households face in the future and effectively solving the three rural issues, which has received extensive attention [1,2,3]. Since the reform and opening-up policy was implemented, China has sequentially formulated and released 20 No.1 documents addressing the “three rural (agriculture, rural areas and farmers)” issues. The five No.1 documents released in 1982–1986 mainly focus on rural reform and agricultural development, including the household contract responsibility system and agriculture serving as the basis of national economy, increasing rural investment, and other rural reform policies. These have effectively promoted the reform of the circulation system of agricultural products and agricultural industrialization. However, the progress of rural reform was still relatively slow, and the income gap between urban and rural areas was widened. The 14 No.1 documents released in 2004–2017 emphasize the extreme importance of the three rural issues in the socialist modernization of China. The formulated policies include the promotion of farmers’ income, the new socialist countryside, the agricultural modernization, the integration of urban and rural development, the supply-side structural reform of agriculture, and many other policies which can benefit the development of agriculture and farmers. These polices have solved many problems in rural development, promoted the construction of new socialist countryside and narrowed the gap between urban and rural areas. The No.1 document released in 2018 proposed to implement the strategy of rural revitalization. It also put forward specific goals for speeding up agricultural modernization and taking a characteristic Chinese road to revitalizing socialist villages. These are the necessary requirements for achieving the common prosperity of all people in China. The above-mentioned No.1 documents reflect the different focus of three rural issues at different stages of development of China. These policies also have great influence on rural livelihoods [4,5,6]. A secure livelihood is the main goal of human activities. By production and consumption activities, humans exert influences on land. Livelihood is thus the major driving force of evolution of human-land systems. Rural households are the basic units and micro business entities for the economic and social development in rural areas. Their livelihoods include the abilities and assets required for survival and development, production activities, and rights to obtain all of these. Since the reform and opening-up policy was implemented, rural households, under the influence of industrialization and urbanization, have had more livelihood choices, which leads to continuous livelihood change [7,8]. This further affects land use process, ecological environment evolution, natural resource management, ecological compensation, and livelihood resource allocation [9,10,11]. Livelihood assets, livelihood strategies, and livelihood outcomes are at the core of rural household livelihoods. Rural households utilize obtainable livelihood assets, select proper livelihood strategies and obtain livelihood outcomes. The amount of livelihood assets that rural households have determines their consumption-type investment and further affects the construction of rural settlements. Livelihood assets, as the basis of livelihood structure, determine the selection of livelihood strategies, thus accurately quantifying the livelihood assets of rural households can help identify the trend of their development [12,13,14,15].

Rural settlement is not only a place where farmers live, but also an important production space. It is a “complex” with multiple functions closely related to rural household livelihoods [16,17,18]. The formation and development of rural settlements is not only a result of households’ diverse selection of livelihoods but also a result of the process where households influence the pattern, form, and structure of rural settlements. Finally, rural settlement land use experiences significant changes and settlements are even re-constructed [19,20]. The form and function of rural houses change greatly with time and livelihoods. At the same time, due to the changes in the spatial pattern and form of rural settlement, farmers also need to change their livelihoods in order to adapt to the new spatial pattern of rural settlement [21,22,23]. For a long time, researchers, restricted by their own disciplines, have either studied household livelihood strategies, quantification of livelihood assets, and livelihood changes from the perspective of regional economics and sustainable development [24], or studied spatial evolution of rural settlements from the perspective of settlement geography [25]. All of them have just studied single problems from single perspectives. There is little research combining both perspectives, which is not conducive to revealing the internal mechanism of rural settlement evolution from multidimensional scale. In fact, the evolution of rural settlements is not only related to natural and social economic factors, but also closely related to the change of farmers’ livelihoods [26]. The spatial-temporal variation of livelihoods leads to difference in rural settlement land use. Current research has mostly focused on the former while ignoring the latter. In addition, most of the research mainly concentrates on county or a larger scale [27], but neglects the most basic unit of rural settlements, i.e., village. Therefore, the research results cannot well guide the village-scale construction.

Rural settlement actually consists of rural households and rural households are the main subjects of economic activities in rural areas. Because of the difference in family economic income and the change of consumption concept, the cognition, demand, and decision making towards residential improvement vary from household to household. The rural residential land use change in the new period is no longer the pure expansion and merging of areas, but reconstruction and integration at the village scale under the influence of farmers’ production and living behavior. This can comprehensively reflect the development of village, agriculture and rural households. In this paper, Sihe village (Jichuan Town, Tongwei County, mid-Gansu loess hilly region, China) was taken as an example. Based on the participatory rural assessment method (PRA), household survey was conducted to analyze the allocation of household assets and livelihood diversification since 1988. We also explored the influence of farmers’ livelihood change on rural settlement land use. The biggest difference between our paper and previous studies lies in that: We analyzed the temporal changes of farmer’s livelihoods and rural settlement land use, constructed a matrix showing the farmers’ livelihood strategy change, explored the process and the key period of livelihood change, and revealed the relationship and response mechanism between livelihood change and rural settlement land use change. The results might provide theoretical reference for the selection of new village location, reconstruction of rural settlements, and creation of livable rural environment.

## 2. Overview of Study Region

**Figure 1:**
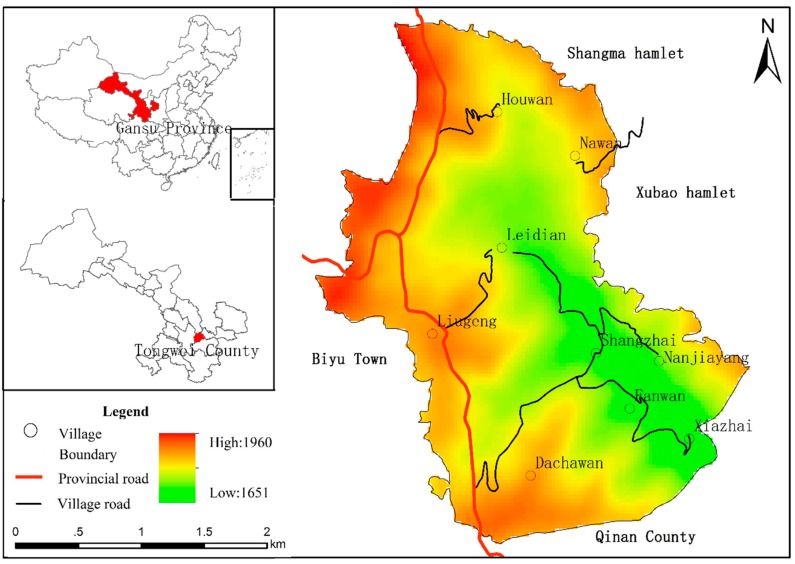
Map of the study area.

Sihe village is located in mid-Gansu loess hilly region, in the western part of Jichuan Town (Tongwei County, Gansu Province, China). It has a total area of 7.71 km^2^, 15 km distant from town area and 25 km distant from county seat. Sihe village is adjacent to Shangma village and Xubao village (Tongwei County) in the northeast, and Wangfu Town (Qinan County) in the southwest. It is located between 105°25′52.97″ E, 35°8′10.21″ N. The elevation of most regions in Sihe village ranges from 1600 to 2000 m, with an average elevation of 1763 m. Sihe village has a warm, moist climate, with enough solar radiation, average annual rainfall of 450 mm, and frost-free period of 150 days [28] (Figure 1).

Sihe village has jurisdiction over nine communities, including Dachawan, Shangzhai, Leidian, Napowan, Houwan, Nanjiayang, Xiazhai, Fanwan, and Liugeng. Among them, liugeng is located on the top of mountain, Shangzhai and Xiazhai are located in valley bottoms, and the remaining communities are distributed on mountain slopes. In 2017, there were a total of 327 households in Sihe village. The total population was 1233, among which 700 were labor force participants. The per capita net income was 3000 Yuan. The total cultivation area was 452.13 hm^2^, with per capita cultivation area of 0.31 hm^2^ [29].

There are three reasons why Sihe villages was selected for investigation: (1) It is a typical poor village. Tongwei County is a poor county of China, and Sihe village is a poor village in Tongwei County. In Sihe village, the scale and mechanization of agricultural land management are relatively low. In addition, the residence is less limited by the farming radius, but it is greatly influenced by the production and lifestyle of farmers; (2) The development context for Sihe village is complete and clear. The development of this villages is closely related to national policies. Since the reform and opening up in 1978, it has been in a relatively stable state of development, with a complete social transformation process; (3) Household livelihoods and evolution patterns of Sihe village are typical and representative. The production and lifestyle of farmers as well as the scale, structure, and morphology of rural settlement land use in Sihe village are universal in mid-Gansu loess hilly region, thus the study of the Sihe village can provide theoretical reference for the construction of other rural settlements.

## 3. Data Sources and Research Methods

### 3.1. Data Sources

Data came from three sources: (1) Basic maps: Topographic map of Sihe village (1:50,000) and vector administrative boundary (1:50,000) in 2010 were obtained from Gansu Province Surveying and Mapping Bureau; (2) Land use vector data: Land use data in 2016 were obtained by UAV aerial photography. Rural settlement plaques were then extracted and referred to the detailed inspection data of land use in the same year for correction; (3) Data came from the rural household questionnaire in field survey based on Participatory Rural Appraisal (PRA). The PRA, a method of rural social investigation, was developed in the early 1990s and has been widely used. The core of this method is to understand the local situation through the communication with local people. In this paper, the field survey based on PRA was conducted as follows: (1) Visited each of the households in Sihe village. With the help of village head and the group leaders of Sihe village, a 16-day household survey was conducted in July and December 2017, with the survey in December as the supplementary survey. A total of 190 effective questionnaires were obtained, meaning that 58.1% of households gave effective responses (80 households had already moved out of Sihe village and 57 households refused the interview, see Table 1). Interviews were conducted after we introduced ourselves, explained our purpose, and gained the residents’ understanding and support. During the interview, information about their family situation, income and expenses, residence, production, behavior willingness, etc. were obtained; (2) Interviewed some special villagers. We mainly selected the following villagers: villagers who were more than 65 years old, village leaders who had worked for many years and did not retire, retired village leaders, and accountants. For the reliability of the data, no less than two people were selected for each type, and a total of 23 people were selected for all the types. The overall situation of the village was known through the insider interview. The interview contents included the distribution and ownership of various land types, related land use policies, the main economic activities in the village, the internal structure, building materials, morphology, and function of rural houses in different periods, and farmers’ investment in their houses; (3) Collected and annotated the data of current land use in the village. Combining with the aerial images and vectorized data of the village in 2016, the main residential areas, roads and other landforms and features were marked on the map. During the interview, we discussed the land use types and distribution on the aerial images with the interviewees, and obtained the changes of the number, position, and area of the residential houses at different time points since 1988. Then, field investigation was carried out; (4) After the interview, the information was revised. Again, insiders were gathered to discuss the land-use status, political and socioeconomic changes in different periods, as well as the process and causes of land-use change over the decades. Several important historical periods were identified and the obtained information was added to the topographic map and the existing land use map. Field investigation was conducted and the map of past-to-present land use was drawn; (5) Posterior assessment. The information obtained from the survey site was analyzed and referred to related materials. Then, the obtained preliminary results were returned to the above mentioned special interviewees for review. Finally, the data used in the study were determined.

### 3.2. Research Methods

#### 3.2.1. Estimation of Livelihood Assets

##### Construction of Index System for Quantification of Livelihood Assets

By quantitative analysis of livelihood assets, the structure and allocation of assets can be intuitively revealed, which can help researchers to determine the stage of development that farmers are in and thus differentiate them [30]. Many frameworks have been proposed to analyze livelihoods. After a comprehensive comparison, we found that the Sustainable Livelihoods Framework developed by the UK’s Department for International Development (DFID) is the most influential one and also has the widest application [31]. In this framework, farmers are considered as subjects that make a living in a vulnerable background. They can use the natural assets, material assets, manpower assets, financial assets, and social assets that they accumulate, obtain beneficial livelihood outcomes and achieve their livelihood goals [32]. Therefore, this work was based on the core ideas about quantification of livelihood assets in the Sustainable Livelihoods Framework. According to the survey results and the actual situation of Sihe village, the index system for quantification of livelihood assets considered four kinds of assets: natural assets, material assets, manpower assets, and financial assets (Table 2).

##### Determination of Weight of Index

The initial data were standardized to eliminate the influence of dimension and magnitude on results. For index with positive effects (i.e., the greater the value of index, the more conducive it is to system development), positive index Formula (1) was used for standardization. For index with negative effects (i.e., the greater the value of index, the less conducive it is to system development), negative index Formula (2) was used for standardization. The formulas are as follows:

Positive index:

(1) $$\;Z_{\mathrm{ij}}=\frac{C_{\mathrm{ij}}-\min(C_{j})}{\max\left(C_{j}\right)-\min(C_{j})}$$

Negative index:

(2) $$\;Z_{\mathrm{ij}}=\frac{\max\left(C_{j}\right){-C}_{\mathrm{ij}}}{\max\left(C_{j}\right)-\min(C_{j})}$$

where C_ij_ and Z_ij_ are the initial value and standardized value of the jth index of ith household, respectively; max{C_j_} and min{C_j_} are the maximum and minimum values of jth index, respectively.

In addition, coefficient of variation method was adopted to determine the weight of index in order to minimize the influence of subjective factors on results.

(3) $$\;\delta_{j}=\frac{D_{j}}{{\bar{Z}}_{j}}$$

(4) $$\;W_{j}=\frac{\delta_{j}\;}{\sum_{j=1}^{n}\delta_{j}}$$

where δ_j_, D_j_, ${\bar{Z}}_{j}$, and W_j_ are the coefficient of variation, mean square error, mean, and weight, respectively.

##### Livelihood Asset Estimation Model

Based on the index of livelihood assets and the weight of index, the output value of each sub asset accumulated by farmers is estimated. The formula is as follows:

(5) $$\;C_{i}=\overset{n}{\underset{j=1}{\sum }}Z_{\mathrm{ij}}W_{j}$$

where C_i_ is the value of certain (natural, material, manpower, and financial) assets of *i*th household.

#### 3.2.2. Livelihood Diversity Index

In order to describe livelihood diversity in Sihe village, an index was introduced. Each of the livelihoods that a household is in involved in was assigned a value (i.e., 1) and then the sum of the values was calculated. For example, if the livelihoods of a household include planting and working outside village, then the livelihood diversity of this household is 2.

(6) $$D=\frac{1}{n}\sum_{i=1}^{n}d_{i}$$

where d_i_ is the livelihood diversity of *i*th household; n is the number of households in the region; D is the livelihood diversity in the region.

#### 3.2.3. Rural Settlement Landscape Pattern Index

According to settlement landscape ecology and research methods on landscape pattern index, rural settlement plaque area (CA), plaque number (NP), average plaque area (MPS) were selected as indexes to quantitatively analyze changes in rural settlement land use scale and expansion [33]. Plaque expansion index (D) and plaque square index (S) were selected to describe the evolution of planar morphology of rural settlement plaques. D refers to the degree of difference between plaque shape and circular shape. S refers to the degree of difference between plaque shape and square shape.

(7) $$\;D=\frac{P}{2\sqrt{\pi \times a}}$$

(8) $$\;S=\frac{0.25P}{\sqrt{a}}$$

where a is the area of rural residence plaque (hm^2^) and P is the perimeter of the plaque (m).

## 4. Results

### 4.1. Changes of Households’ Livelihood Assets

#### 4.1.1. Changes in Livelihood Asset Structure

**Figure 2:**
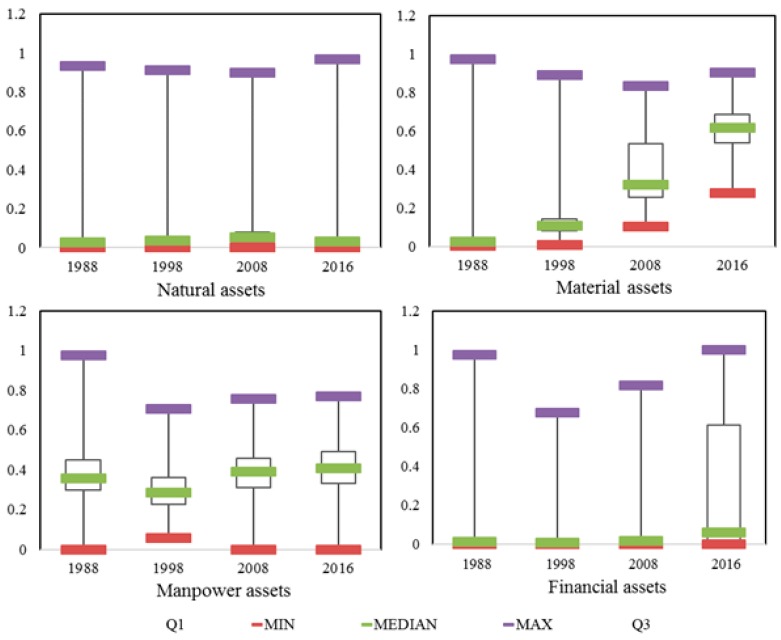
Map of the livelihood asset values of households in Sihe village.

According to Formula (5), the values of four kinds of assets were calculated for each household (Table 3, Figure 2). From 1988 to 2016, the average livelihood asset value per household increased from 0.48 to 1.288, with an annual increase of 0.049. Note that this value increased more rapidly from 2008 to 2016. The average natural asset value per household increased from 0.035 in 1998 to 0.084 in 2008 and then decreased to 0.046 in 2016. The average material asset value per household increased continuously from 0.039 in 1998 to 0.606 in 2016, with an annual increase of 0.021. The average manpower asset value per household and the average financial asset value per household showed the same trend of change. The former decreased from 1988 to 1998 and increased from 1998 (0.295) to 2016 (0.412). The later also decreased from 1988 to 1998 and increased from 1998 (0.025) to 2016 (0.224). Notably, the increase in the average financial asset value per household from 2008 to 2016 was significant and accounted for 93% of that from 1988 to 2016.

**Figure 3:**
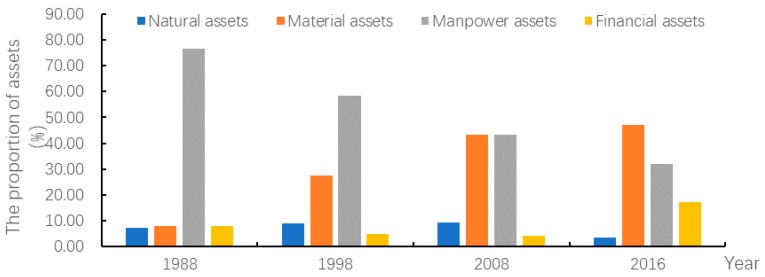
The livelihood asset structure in 1988, 1998, 2008, and 2016.

The livelihood asset structure varied significantly with time and the difference among the values of four assets was gradually reduced (Figure 3). In 1988, there was a great difference in value among four assets. The value of manpower assets accounted for the highest proportion (77%) of the total value of livelihood assets. The values of the other three assets were close to each other, accounting for less than 10% of the total value. In 1998, the value of manpower assets still accounted for the largest proportion (59%) of the total value of livelihood assets. The proportion of material asset value increased greatly from 8% in 1988 to 27% in 1998. The value of financial assets still accounted for the smallest proportion (4%) of the total value of livelihood assets. In 2008, significant changes occurred in livelihood asset structure. The proportion of material asset value greatly increased to 43.16% and was close to that of manpower asset value. The value of financial assets still accounted for the smallest proportion of the total value of livelihood assets. In 2016, the difference in proportion among four assets still existed but was significantly reduced compared with that in 1988. The value of material assets accounted for a larger proportion (reached 47%) than that of manpower assets. The value of financial assets accounted for 17% of total value. The value of natural assets accounted for the smallest proportion (4%) in the total value.

The allocation of assets among households varied with time (Table 4, Figure 2). The difference in natural assets among households was large, while the difference in manpower assets among households was small. These differences also varied with time. From 1988 to 1998, the allocation of four assets among households changed little. In both periods, there was relatively great difference in natural assets among households, whereas there was small difference in the other three assets among households. The value of manpower assets owned by more than 60% households was smaller than 0.4. The value of financial assets owned by more than 75% of households was smaller than 0.02. The difference in material assets among households was the smallest. The value of material assets owned by 100% and 96% households was smaller than 0.4 in 1988 and 1998, respectively. From 1998 to 2008, the allocation of four assets among households changed significantly. The difference in natural assets among households reduced and the value of natural assets owned by more than 66% of households was larger than 0.4. However, the difference in the other three assets among households increased. The proportions of households with material asset value and manpower asset value smaller than 0.4 decreased from 96% and 78% in 1998 to 58.94% and 56% in 2008, respectively. The difference in financial assets among households was the largest. The values of financial assets owned by 39% and 13% of households were smaller than 0.01 and larger than 0.04, respectively. In 2016, the allocation of four assets among households changed significantly compared with that in 1998. There was great difference in all asset types, except for financial asset, among households. The proportion of households with financial asset value greater than 0.4 increased from 13% in 2008 to 65% in 2016. In sum, 2008 was a key year with significant changes in allocation of livelihood assets among households. In this year, the difference in natural assets among households reduced, while the difference in the other three assets among households increased.

#### 4.1.2. Changes in the Spatial Pattern of Households’ Livelihood Assets

**Figure 4:**
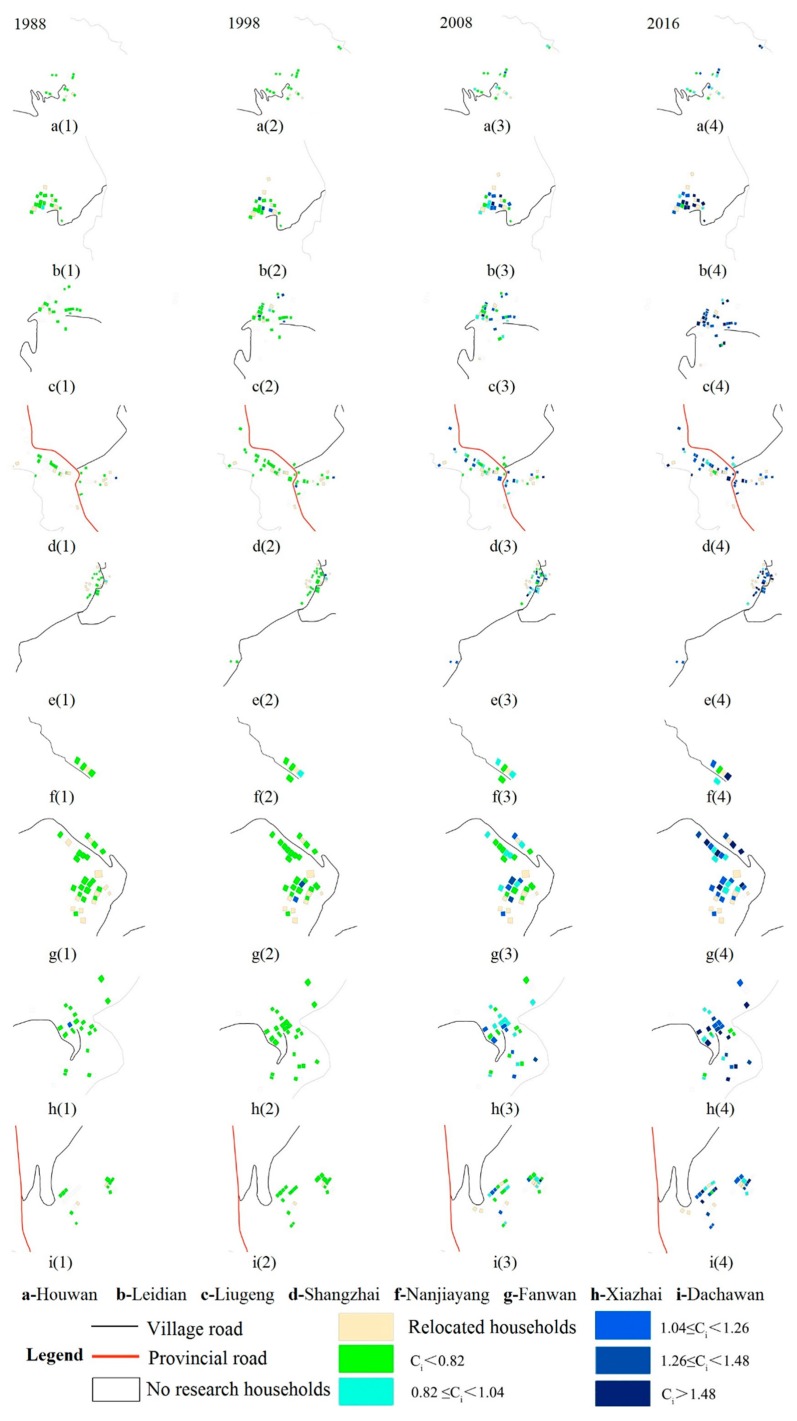
Spatial variation of households’ livelihood assets in 1988, 1998, 2008, and 2016.

Figure 4 shows the spatial distribution of livelihood assets in Sihe village. In 1988, the livelihood asset values of all households were smaller than 0.82, thus there was small spatial variation of livelihood assets in Sihe village. In 1998, as household livelihoods became more diverse, there occurred spatial variation of livelihood assets. Large livelihood asset values appeared in five communities including Liugeng, Fanwan, Shangzhai, Leijiadian, and Napowan, mainly in the middle of Sihe village with low elevation (Figure 1 and Figure 4). In 2008, significant changes occurred in the spatial pattern of livelihood assets in Sihe village. Large livelihood asset values appeared in the north part of the village, while low livelihood asset values appeared in the south part. Napowan in the north part and Shangzhai in the middle part experienced significant change in spatial patterns of livelihood asset values. In these two regions, households with high livelihood values became more concentrated. Houwan and Nanjiayang experienced little change in spatial pattern of livelihood asset values. In other communities, households with different livelihood asset values were dispersedly distributed. In 2016, many zones were characterized by high livelihood asset values. In the north part, Napowan, Leidian, and Shangzhai were zones with high livelihood asset values that were close to each other. There were only a few households with livelihood asset values lower than 1.04. In the south part, Liugeng, Xiazhai, Fanwan, and Dachawan were also high value zones, though some households with low values were distributed in these zones. Houwan, in the northmost part, however, was a zone with low values and there were only a few households with livelihood asset values higher than 1.04. As the micro subjects of economic and social activities in rural areas, households adjust their own livelihood needs under the influence of external environment, and spatial variation of livelihood assets thus occurs. Further, difference in livelihood strategy and livelihood development occurs. Some households enter the agricultural sector, while others enter non-agricultural sectors.

### 4.2. Household Livelihood Diversity

Field investigation results revealed that the livelihood activities of farmers in Sihe village include planting, fruiting, breeding, going out to work, doing business, etc. Livelihood diversity varied with time and tended to increase (Table 5). In 1988, most households depended on few livelihoods. For example, the number of households relying on only 2 livelihoods reached 108, accounting for 59% of all households investigated. Households relying on only 1 livelihood and 3 livelihoods accounted for 21% and 21% of all households investigated, respectively. However, the number of households relying on more than 3 livelihoods was 3, accounting for only 2% of all households investigated. In 1998, livelihood diversity increased. Households relying on 3 or more livelihoods accounted for 55% of all households investigated. There were only 12 households relying on 1 livelihood, accounting for 6.31% of all households investigated. In 2008, livelihood diversity further increased. Households relying on 3 or more livelihoods accounted for 75% of all households investigated. The proportion of households relying on no more than 2 livelihoods decreased to 25%. The number of households relying on 1 livelihood was as low as 6. In 2016, households relying on 3 or more livelihoods accounted for 77% of all households investigated. The number of households relying on 4 or more livelihoods reached 50, accounting for 26% of all households investigated. The livelihood methods of farmers in Sihe Village include planting, fruiting, breeding, going out to work, doing business, etc.

The livelihood diversity indexes for Sihe village and for communities in the village were calculated (Table 6). Livelihood diversity index in Sihe village increased from 2.01 in 1988 to 3 in 2016, suggesting more diverse livelihoods. Among the nine communities, Dachawan, Fanwan, and Xiazhai experienced decreases in livelihood diversity index from 2008 to 2016. The livelihood diversity indexes of other communities increased continuously from 1988 to 2016. In 1988, livelihood diversity in the nine communities were low. Fanwan and Shangzhai were characterized by the highest and lowest livelihood diversity indexes, respectively, which were 2.29 and 1.83, respectively. In 1998, livelihood diversity indexes of all communities increased to be higher than 2. Nanjiayang and Xiazhai were characterized by the highest and lowest livelihood diversity indexes, respectively, which were 3 and 2.3, respectively. In 2008, livelihood diversity in the communities changed only a little compared with that in 1998. The livelihood diversity indexes of Nanjiayang, Dachawan, and Fanwan exceeded 3. In 2016, livelihood diversity in the communities changed significantly. All communities, except for Houwan and Xiazhai, had a livelihood diversity index higher than 3. The livelihood diversity index of Nanjiayang even reached 3.25.

### 4.3. Household Livelihood Change

**Figure 5:**
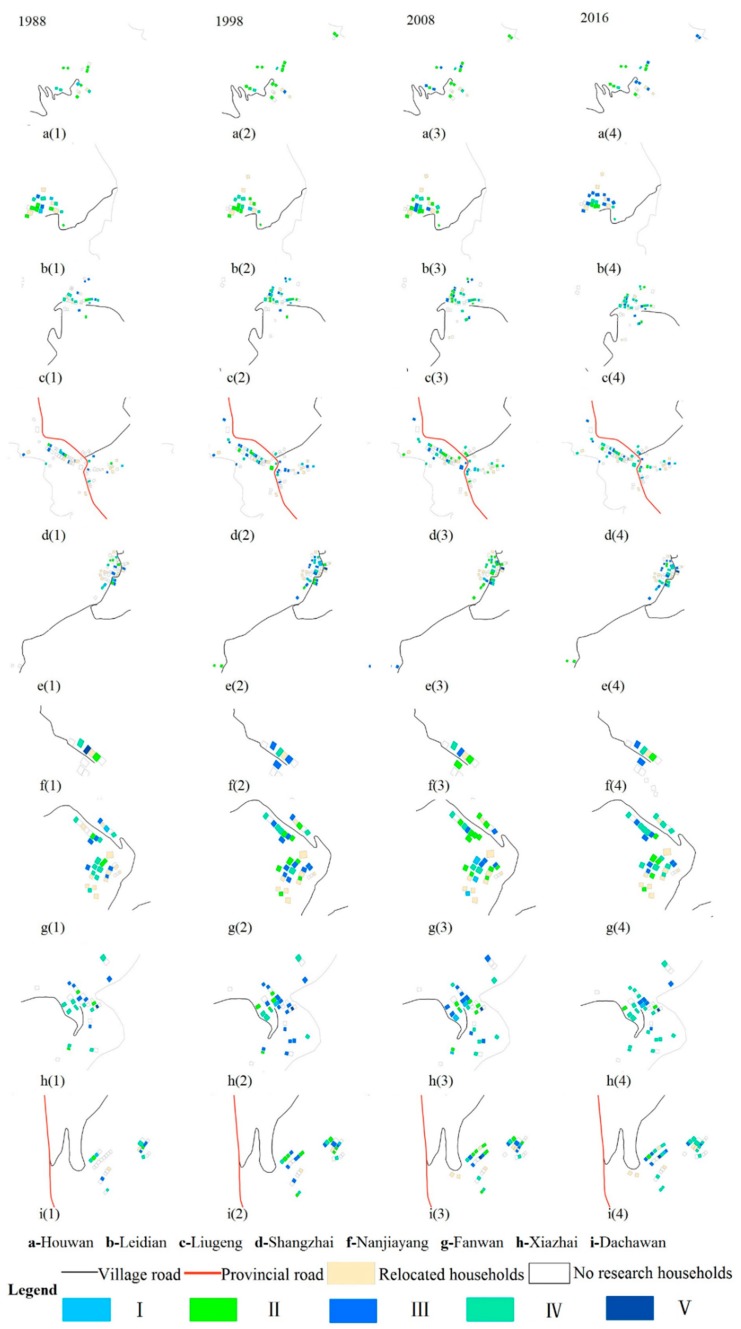
Spatial distribution of different types of households in 1988–2016.

Households’ livelihood change refers to the fundamental change in the occupation or business that households rely on. It is a process of farmers’ dependence on agricultural production on rural land gradually reducing [34]. By analyzing the livelihood assets and livelihood diversity in Sihe village in 1988–2016, we can see that natural assets and the other assets have opposite effects on households’ livelihood development. Also, the difference in natural asset value among households was the greatest. Therefore, households were classified into five groups mainly according to their natural assets (Table 7 and Figure 5). The five groups include: Agricultural production group (I), Diverse agricultural production group (II), Agricultural and non-agricultural production group (III), Diverse non-agricultural production group (IV), and Non-agricultural production group (V). The number of households in group V remained the smallest in four periods, only 2 in 1988 and 1998, and 3 in 2008 and 2016, accounting for 2% of all households investigated. The number of households in group I was the second smallest and decreased with year. It decreased to 11 in 2016, accounting for 6% of all households investigated. These households in group I all managed a relatively large area of land, all area exceeds 2.3 hm^2^. The scale of agricultural production was thus relatively large. The number of households in group II increased from 44 in 1988 to 81 in 2008, but further decreased to 33 in 2016. These households managed a limited area of land and their other asset values were lower than the corresponding average values. The lack of the other three assets limited livelihood change. In the future, if no relevant policies are formulated and implemented, these households will still engage in traditional agricultural production. They will plant various crops and raise livestock/poultry to fulfill their survival and living needs. Thus, the agricultural production activities tend to be diverse. The number of households in group III fluctuated and reached its maximum (71) in 1998. The various assets of these households were at average levels and enabled them to engage in non-agricultural production. They relied on agricultural production to secure the necessities of life and then might actively engage in non-agricultural production. The number of households in group IV decreased first and then increased. The manpower, material, and financial assets of these households were more than the corresponding average assets. These households had few worries about their lives and developed toward the secondary and tertiary industries. However, since their development into non-agricultural sectors is still in an initial stage, inter-industry mobility should be high and the non-agricultural production activities tend to be diverse. Since household contract responsibility system was implemented in Sihe village in 1984, farmers have been actively involved in production activities. Restricted by agricultural production conditions, many households engage in diverse agricultural production activities at the same time or engage in non-agricultural production activities. As time goes by, this trend tends to be more significant.

We further obtained the matrix showing changes in household livelihoods (Table 8). The trend of change in household livelihoods was different in different periods. From 1988 to 1998, more households tended to engage in agricultural production and the agricultural livelihoods tended to be more diverse. Livelihood change trend of IV (Diverse non-agricultural production) → III (Agricultural–non-agricultural production) → II (Diverse agricultural production) → I (Agricultural production) was observed (bottom left corner, Table 8). The trend of IV → III → II was especially significant. About 47% households originally in group IV turned into households in groups III and II. On the other hand, 11 households (61%) originally in group I turned into households in group II, suggesting that agricultural livelihoods tended to be diverse. The period 1998–2008 was a transition period in which both trends of livelihood change toward agricultural production and non-agricultural production were significant. Twenty-two households (52%) originally in group IV turned into households in groups III, II, and I. Thirty-six households (50.70%) originally in group III turned into households in groups II and I. On the other hand, 12 households (71%) originally in group I turned into households in groups II, III, and IV. Seventeen households (29%) originally in group II turned into households in group III. Twenty-three households (32%) originally in group III turned into households in groups IV and V. From 2008 to 2016, the trend of livelihood change toward non-agricultural production was significant and the non-agricultural livelihoods tended to be more diverse. Specifically, a trend of I → II → III → IV was observed. About 88% households originally in group I turned into households in groups II, III and IV. About 74% households originally in group II turned into households in groups III and IV (35 and 25 households, respectively). About 51% households (24) originally in group III turned into households in group IV.

### 4.4. Rural Settlement Land Use Change in Response to Household Livelihood Change

Rural households, as rational “economic men”, always change their livelihoods according to changes in internal and external environments in order to maximize their economic profits. Rural houses, as “complexes” with multi functions, are places where farmers live and production activities occur. Farmers adjust the size, structure, and morphology of their houses to adapt to the changes in their livelihoods.

#### 4.4.1. The Response of Rural Settlement Land Use Scale

Land-use maps for Sihe village were extracted and characteristic land use indicators were obtained (Table 9). These indicators changed significantly from 1998 to 2008 and remained stable from 2008 to 2016. Total area of rural settlement plaques increased from 8.75 hm^2^ in 1988 to 10.23 hm^2^ in 2008, with an annual increase of 0.074 hm^2^. The number of rural settlement plaques increased from 305 in 1988 to 354 in 2008, with an annual increase of 2.45. After 2008, both indicators changed little. In Sihe village, land use expansion scale and speed in different periods followed the order: 1988–1998 > 1998–2008 = 2008–2016. This illustrated that as China gradually promoted the conversion of residential land into cultivation land, the expansion of residential area in Sihe village experienced a transition from pure expansion to coexistence of expansion and shrinkage. From 1988 to 2016, the average area of rural settlement plaques changed only a little and basically remained stable. The average area was 286.84 m^2^ in 1988, decreased (by only 0.52 m^2^) to 286.32 in 1998, and increased to 288.88 m^2^ in 2008. The areas of the largest and smallest plaques changed little from 1988 to 2016 and remained 890.16 m^2^ and 79.88 m^2^, respectively.

At community scale, all communities, except for Fanwan, experienced an increase in total area of rural settlement plaques. Specifically, the total area of rural settlement plaques in Dachawan, Liugeng, Leidian, Houwan, Napowan, and Xiazhai increased first and then remained stable after 2008. The total area of rural settlement plaques in Nanjiayang and Shangzhai remained unchanged after 1998. The changing trend of the number of rural settlement plaques was basically the same as that of the total area of rural settlement plaques. The average areas of rural settlement plaques in Dachawan, Leidian, and Houwan increased first and then decreased. This indicated a transition of residential area expansion from distributed expansion to central expansion. The expansion of residential areas in Nanjiayangpo, Shangzhai, and Xiazhai was mainly the distributed type.

The average rural settlement area per household for different household groups (I–V) was calculated (Table 10). The average rural settlement areas per household all exceeded 200 m^2^ and almost all exceeded the average homestead area standard (225 m^2^) set by Gansu government. In 1988, the average rural settlement area per household was the largest for group I (302.76 m^2^) and the smallest for group V (224.55 m^2^). 2008 was a year before and after which the average rural settlement areas per household for all groups of households showed opposite change trends. The average rural settlement areas per household for groups I and II decreased slightly from 1988 to 2008 and then increased a little from 2008 to 2016. The average rural settlement areas per household for groups III–V increased from 1988 to 2008 and then decreased from 2008 to 2016. Notably, the average rural settlement area per household for group V increased significantly (by 77.71 m^2^) from 1988 to 1998. During field investigation, it was found that homestead areas in Sihe village had remained almost unchanged since the reform and opening-up policy was implemented in 1978. Only a few farmers constructed houses in new sites and most farmers reconstructed their houses in original house sites. In addition, the four-year mean of the average rural settlement area per household for group I was as high as 298.55 m^2^. The four-year mean of the average rural settlement area per household for different groups followed the order: I > II > III > IV except group V. This illustrated that livelihood change was the main reason for changes in the scale of rural settlement land use. For households relying on agricultural production, a large area of land was used for residence, the average rural settlement area was 298.55 m^2^. As households engaged in various agricultural production activities, the area of land used for residence was decreased. When households mainly relied on non-agricultural production, they decreased their investment in agricultural production and reduced the area of land used for storage, livestock/poultry raising, etc. Therefore, the rural settlement land use scale for these households was small. Group V has the largest average rural settlement area among the five groups. Notably, there were only 2 households in group V in 1988–1998. The average rural settlement area increased significantly in 1998, which is closely related to the New rural construction policy. In 2008, there were 3 households in group V, and the average rural settlement area was 303.97 m^2^. After 2008, the average rural settlement area changed little. Since the number of rural households in this group was small and the households in this group remained almost the same from 1988 to 2016, their livelihoods have little to do with the rural settlement land use scale.

#### 4.4.2. The Response of Rural Settlement Land Use Structure

**Figure 6:**
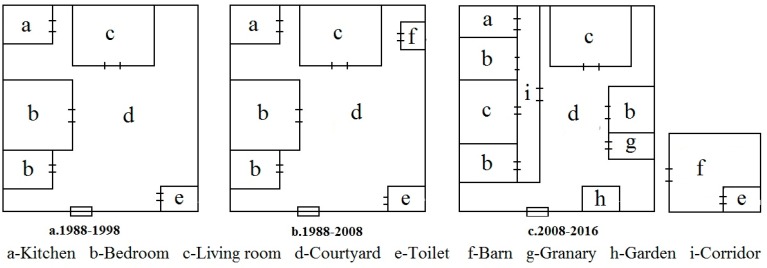
Changes in rural settlement land use structure in Sihe village.

From 1988 to 1998, the implementation of household contract responsibility system promoted farmers’ enthusiasm in agricultural production. With increase in agricultural inputs such as pesticides, fertilizers, and machinery, land productivity was significantly improved, the grain yield per hectare is increased from 974.85 kg to 1473.98 kg. At the same time, with the progression of industrialization, farmers began to seek non-agricultural livelihoods, thus livelihood diversity increased. The livelihoods in Sihe village mainly included planting crops, raising livestock/poultry, and working outside the village. In order to adapt to the farmers’ livelihoods, the houses in Sihe village had a structure of “bungalow + courtyard”. Farmers planted vegetables and raised livestock/poultry in their yards. In this period (1988–1998), the houses also had agricultural production function (Figure 6a). From 1998 to 2008, under the enhanced influence of industrialization and urbanization, household livelihoods mainly included crop planting and working outside village. A large portion of labor force was transferred to non-agricultural sector. In response to non-agricultural economy, household livelihoods changed significantly. The courtyard was paved with concrete or other materials so that food can be sun dried on its hardened surface. Farmers still raised livestock or poultry, thus barn was built in their courtyards (Figure 6b). Such agricultural production activity could help improve farmers’ income. After 2008, increased livelihood diversity and livelihood change toward non-agricultural sector have led to good livelihood outcomes. Farmers increase their investment in house construction. The requirement for the quality of living environment also becomes higher. Although the house structure of “bungalow + courtyard” remains unchanged, new living space and leisure space (such as family garden) are built in the yard (Figure 6c). In this period, the house is only a place where people live, and its functions do not include agricultural production. A barn is built outside the house. In this way, the living environment is improved and the residential area is also increased at the same time.

There was no obvious difference in rural settlement land use structure among groups I–IV. The field investigation revealed that it often took a long time for farmers to accumulate wealth and reconstruct their houses. The adjustment of rural settlement land use structure thus lagged behind household livelihood change. In addition, the uncertainty in non-agricultural production made it hard for households to totally give up agricultural production. Although they might temporarily not engage in agricultural production, lands used for various purposes such as crop planting were still kept [35].

#### 4.4.3. The Response of Rural Settlement Land Use Morphology

The morphology indexes of rural settlement plaques in Sihe village in different periods were calculated (Table 11). At the village scale, the expansion index D and square index S of rural settlement plaques decreased from 1988 to 1998. After 2008, they increased a little and then remained stable. This suggested that the two-dimensional morphology of rural settlement plaques was relatively regular before 2008 and become more irregular (deviated from square shape or circular shape) after 2008. In terms of livelihood strategy, D and S of residence plaques for groups I, III, and V decreased from 1988 to 1998, increased from 1998 to 2008, and then decreased again. This indicated that the morphology of rural settlement plaques fluctuated. From 1998 to 2008, the morphology deviated from square shape or circular shape, but in general it changed little. D and S of rural settlement plaques for group II increased from 1988 to 2008 and then decreased. This illustrated that the morphology of rural settlement plaques became closer to circular shape or square shape after 2008. D and S of rural settlement plaques for group IV decreased from 1988 to 1998 and then increased. This showed that the morphology of rural settlement plaques gradually became more irregular and deviated from circular shape or square shape. In sum, household livelihood changes had influence on the morphology of rural settlement plaques. For households relying on few livelihoods, the changes in the morphology of rural settlement plaques was characterized by a trend of “regular → irregular → regular”. For households relying on diverse livelihoods, the changes in the morphology of rural settlement plaques was characterized by a trend of “regular → irregular”.

## 5. Discussion

With the progression of industrialization and urbanization, the works and working locations of farmers have changed significantly. The production activities of rural households in China gradually change from agricultural–non-agricultural production to non-agricultural production and in-situ non-agricultural production. When households engage in the former, they “leave the land but not the village” in certain season when non-agricultural production occurs. When households engage in the latter, however, they “leave the land and the village”. In rural areas of China, farmers’ ways of production, lifestyles, and ways of communication become more “social” [36]. Rural settlements are places where villagers live and engage in production activities. The scale, structure, and morphology of rural settlement land use gradually change as livelihoods change and social economy develops [37].

### 5.1. Comparison of Livelihood Assets Evaluation Models

Many frameworks have been proposed for livelihood analysis, such as the analytical framework developed by the Department for International Development (DFID), the United Nations Development Programmed (UNDP), and Care International (CARE) [15,38]. However, the most widely used framework is the analytical framework developed by DFID, that is, the SL framework [38]. This framework provides the main idea for the study of farmers’ livelihoods here, and it is helpful to clarify the intricate relationship among many factors affecting farmers’ livelihoods. It considers farmers as subjects that make a living in a specific vulnerable background. In this context, livelihood assets are the key and core of the whole livelihood analysis framework. The nature and structure of livelihood assets determine the livelihood strategies adopted by the farmers, and different livelihood strategies will lead to different livelihood outcomes. Meanwhile, the livelihood outcomes have counterproductive effects on livelihood assets, which in turn determine the nature and structure of livelihood assets [30].

The framework developed by DFID provides a good way of livelihood analysis. It provides guidance for the study of livelihoods and poverty and identification of the core factors that affect livelihoods as well as the links and interactions among all factors [19]. On this basis, targeted measures can be designed. The framework can be applied to different scales, such as countries, regions, small basins, villages, families, and individuals, and it is conducive to improving our understanding of the livelihoods of people, especially poverty-stricken or marginalized groups [39].

### 5.2. Household Livelihood Change Promoted by the Development of Social Economy

The structure and spatial distribution of livelihood assets determine the resource advantage and spatial possibility of household livelihood development [40]. The differentiation of rural households is mainly restricted by the development of local social economy and other external factors. Changes of rural household livelihoods will lead to changes of farmers’ land use concepts and behaviors, and further influence land use and economic development at village scale or even a larger scale [41]. For Sihe village, 2008 was a key year of livelihood change. The livelihood asset structure, livelihood diversity index, and livelihood strategy changed oppositely before and after 2008. In general, livelihood change in Sihe village was driven by two aspects: (1) changes of farmers’ ideas under the development of local social economy and other external environmental factors (2) the implementation of national and local rural development policies. When different policies were implemented, rural areas developed towards different directions and farmers had different ways of production and lifestyles, which directly affected their livelihoods.

Since the reform and opening-up policy were implemented in 1978, the reform of household contract responsibility system has proposed, the central No.1 document on the theme of “three rural” which has been released from 1982 to 1986 for five consecutive years, has promoted the rapid growth of agriculture for more than 10 years, greatly supported the development of China’s economy, promoted farmers’ enthusiasm in agricultural production. With increase in agricultural inputs such as pesticides, fertilizer, and other production factors, land productivity has been significantly improved. At the same time, with the progression of industrialization, rural households begin to seek non-agricultural livelihoods. With the improvement of farmers’ material living standards, agricultural livelihoods are unable to satisfy their various consumption needs. Driven by both higher income and more benefits, some farmers start to seek new livelihoods, so the livelihood diversity and livelihood assets are gradually increased. In the mid and late 1980s, the focus of national reform shifted from rural areas to cities, opening up the new era of China’s urban economy development and having a huge impact on the countryside. From 1988 to 1998, increase in households’ livelihood assets was due to improved livelihood diversity and more to increased manpower assets. In this period, both the number and education level of labor force participants were increased. This suggested the importance of population size and quality in this period. In the late 1990s, the impact of urbanization and industrialization on agriculture was aggravated, farmers’ enthusiasm for grain was greatly frustrated, the increase of farmers’ income was slowing down, and the income gap between urban and rural residents continued to expand. In the six years from 1997 to 2003, the income ratio of urban and rural residents increased from 2.47 to 3.23. Therefore, the state has made great efforts to adjust the strategy of urban and rural development and the policy of guidance. After 18 years, the theme of Document No.1 of the Central Committee, which began in 2004, returned to the three rural areas again, and thus has been continued to the present. Under the influence of various national policies and socialist market economy, agricultural production has been gradually mechanized and there has been surplus labor force in rural areas of China, farmers’ concepts about living and production change continuously. As farmers’ response to industrialization and urbanization become stronger, some of them give up raising livestock and poultry and transfer to non-agricultural sectors. Meanwhile, with increase in income, farmers have higher requirements for production and living conditions. Part of their income is spent on home improvement, purchasing of electrical appliances and production tools, etc. Therefore, farmers’ material assets have increased and basically remained stable after 2008.

### 5.3. Rural Settlement Land Use Change Driven by Household Livelihood Change

Rural settlement is a place where farmers live and engage in production activities. With changes in livelihoods, the scale, structure, and morphology of rural settlement land use have also changed [25]. Under the comprehensive influences of internal and external factors, farmers change the size, structure, and morphology of their houses to adapt to their livelihood changes [42]. These changes can lead to changes of rural houses’ functions and eventually lead to changes in rural settlement land use [43]. From 1988 to 1998, farmers mainly relied on agricultural livelihoods. In order to adapt to their livelihood needs, farmers planted vegetables and raised livestock/poultry in their yards and places around their residence. By engaging in various agricultural production activities, farmers tried to maximize their income. As a result, there was continuous expansion of rural settlement areas and the land use efficiency was relatively low. In addition, under the influence of family concepts and living habits, some families with a large size were gradually divided into families with a smaller size. Therefore, new houses needed to be built and the number of rural settlements increased. After 2000, with changes in agricultural production activities, the size and internal structure of farmers’ houses also changed greatly. The yard was paved with concrete or other materials so that food could be sun dried on its hardened surface. Barns in yards were changed to storage rooms to store food and agricultural machinery. Temporary barns were built outside the yard. When the prices of livestock products were high, farmers might raise livestock in their barns. Such temporary barns can be demolished whenever necessary and then the land can then be used for other purposes. In this period, although the homestead area decreased, the area of land used for production activities did not decrease.

The morphology of rural houses also changes with changes in livelihoods. Improved livelihood diversity and non-agricultural livelihoods result in good livelihood outcomes, thus farmers have higher ability to invest in house construction [44]. In villages close to urban areas, non-agricultural economy is developed. Since only a limited area of land for non-agricultural uses is allocated to farmers, they build new space in their yards or around their homes for non-agricultural production. Therefore, their homes are not only places where they live, but also places where production activities occur. However, if farmers find a job in nearby towns or cities, non-agricultural production activities will not occur in their homes, which will then become places in which they live. As can be seen, the morphology of houses is changed by farmers so that the functions of the houses can adapt to the changes in livelihoods.

During field investigation, we found that the size, structure, and morphology of farmers’ houses are not simply determined by their livelihoods, but also affected by geographical environment, cultural customs, national policies, etc. [45]. However, farmers can actively change the structure and morphology of their houses to adapt to the changes in livelihood needs and thus change the functions of their houses. This is an important perspective from which we can study rural household livelihood changes and their influence on the morphology and functions of rural houses. It is also a perspective from which we can study changes in rural settlement land use and provide guidance for rural construction.

### 5.4. Household Livelihoods and Reconstruction of Rural Settlements

Zhang thought that the rapid urbanization in China has led to great changes in economic space, social space, and settlement space in rural areas, and rural reconstruction in China should focus on the reconstruction of rural space system [46]. Mid-Gansu loess hilly region is characterized by fragile ecological environment, shortage of land for cultivation, and low land use efficiency. Livelihood changes promote changes in rural settlement land use, and phenomena such as rapid random expansion of residential areas, rural hollowing, and idle houses occur, which greatly limit the sustainable development of rural settlements [47]. Rural houses, as the places where farmers live and engage in production activities, are closely related to the actual livelihood needs of farmers. Changes in farmers’ livelihoods can promote changes in the morphology and functions of rural houses and further lead to changes in rural settlement land use, which has become an important reference for new rural construction [6]. Therefore, in the background of farmer differentiation and livelihood change, the structures and morphology of rural houses should be adjusted according to the livelihood characteristics and needs of farmers and finally the goal of rural settlement adjustment can be achieved. This is also the area that we will further investigate in depth.

Based on the relationship between rural livelihood change and land use change, the way and pattern of rural reconstruction should be explored and institutional innovation should be promoted to support rural reconstruction, which is a typical example of combination of rural reconstruction theory and practice [48]. With livelihood changes, farmers’ lifestyles and ways of production also change, which further promote changes in rural society and living space. Rural revitalization has become one of the major driving forces of rural settlement reconstruction. In mid-Gansu loess hilly region, rural settlement reconstruction should be based on farmers’ livelihood assets and needs of livelihood change. It should not only satisfy the living needs of farmers but also consider the overall living condition in the village. In addition, rural settlement reconstruction should consider both housing construction in the past and farmers’ residence willingness and preferences in the future [15]. By rural settlement reconstruction based on household livelihoods and national policies, phenomena such as rural hollowing can be greatly reduced and residential land use efficiency can be improved.

## 6. Conclusions

The structure and spatial distribution of rural households’ livelihood assets are closely related to the changes in rural settlement land use. By analyzing the structure of livelihood assets and their influence on rural settlement land use, we can identify household livelihood changes under the stimulus of external environment. Besides, we can reveal the actual needs of different types of households and the response of rural settlement land use. The research results can promote the intensive use of land for rural settlements and provide a reliable theoretical basis for rural revitalization.

(1) From 1988 to 2016, the average livelihood asset value per household increased from 0.48 to 1.288, with an annual increase of 0.049. Notably, the average livelihood asset value per household increased more rapidly in 2008–2016 than in other periods. The livelihood asset structure varied significantly with time and the difference in proportion among four kinds of assets was gradually reduced. The allocation of livelihoods assets among households varied with time. In general, the difference in natural assets among households was large, while the difference in manpower assets among households was small. These differences also changed with time.
(2) The livelihoods in Sihe village varied with time. The livelihood diversity index increased from 2.01 in 1988 to 3 in 2016. In 1988, many households relied on few livelihoods. There were 108 households relying on two livelihoods, accounting for 59% of all households investigated. After 1988, livelihood diversity increased. The proportion of households relying on three or more livelihoods reached 78%.
(3) 2008 was a key year of livelihood change. The trend of livelihood change was different before and after 2008. From 1988 to 2008, livelihoods changed toward the agricultural sector and agricultural livelihoods tended to be diverse. Specifically, a trend of IV → III → II → I was observed. From 2008 to 2016, livelihoods changed toward non-agricultural sector and non-agricultural livelihoods tended to be diverse. Specifically, a significant trend of I → II → III → IV was observed.
(4) Farmers changed the size, structure, and morphology of their houses so that the houses could adapt to the livelihood changes. The indicators of rural settlement land use scale changed significantly from 1988 to 2008. The total area and number of rural settlement plaques remained stable after 2008. The houses in Sihe village had a basic structure of “bungalow + courtyard” and this basic structure changed little from 1988 to 2016. However, farmers could actively change the internal structure and morphology of their houses to adapt to their livelihood needs. This led to changes in the function of rural houses and finally resulted in rural settlement land use. Livelihood changes have influence on the spatial morphology of rural settlement plaques. For households relying on few livelihoods, the spatial morphology of rural settlement plaques changed toward “regular → irregular → regular”. For households relying on diverse livelihoods, the spatial morphology of rural settlement plaques changed from “regular → irregular”.
(5) Livelihood changes were driven by two aspects: changes of farmers’ ideas under the development of local social economy and other external environmental factors; national and local rural development policies. On this basis, the size, structure, and morphology of rural houses changed and finally rural settlement land use changed. Therefore, the structure and morphology of rural houses should be adjusted according to farmers’ livelihood characteristics and needs, and finally the goal of rural settlement adjustment can be achieved.

## Figures and Tables

**Table 1:** Household survey of Sihe Village.

Community Name	The Number of Total Households	The Number of Surveyed Households	The Ratio of Surveyed Households to Total Households (%)	The Number of Unsurveyed Households	The Number of Households That Moved Out of Village
Dachawan	38	22	58	16	10
Shangzhai	47	24	51	23	9
Xiazhai	34	27	79	7	4
Fanwan	34	24	71	10	7
Leidian	38	25	66	13	3
Napowan	21	15	71	6	5
Nanjiayang	14	4	29	10	8
Liugeng	75	36	48	39	22
Houwan	26	13	50	13	12
Total	327	190	58	137	80

**Table 2:** Index system for quantification of livelihood assets.

Livelihood Assets	Index	Weight	Index Definition
Natural assets	Cultivation area (hm^2^)	0.08	The total area of cultivated lands managed by a household.
	Garden area (hm^2^)	0.92	The total area of gardens managed by a household
Material assets	Home appliance (piece)	0.25	The total amount of motorcycles, refrigerators, washing machines, televisions, and other home appliances
	Production tools (piece)	0.29	The ratio of the amount of production tools owned by a household to the total amount of productions tools investigated (mowing machine, rotary tiller, thresher, pedicab and store).
	Road (m)	0.21	The vertical distance between farmer’s house and the nearest road in the village.
	House	0.25	The quality of house is classified into five grades: good (5), relatively good (4), fair (3), poor (2), and dangerous (1). The classification of the quality of house was in accordance with the “Planning of the construction of Sihe village”, which includes the evaluation of the quality of house.
Manpower assets	The labor ability of household	0.28	The labor ability of each member in the family is assigned a value and then the sum of the values is calculated. No labor ability (0): children aged 0–10 years and people older than 64 years; Limited labor ability (1): teenagers aged 11–18 years and people aged 60–64; Full labor ability (2)
	Vocational skills of family members	0.40	The vocational skills of each member in the family is assigned a value and then the sum of the values is calculated. Non-labor force (0); Agricultural laborer (1); Non-agricultural laborer (2); Working outside village (3)
	Level of education (year)	0.32	The average years of education that family members received
Financial assets	Household’s income (Ten thousand yuan)	0.40	Average annual household income
	Household’s loan	0.60	With loan (1); No loan (0)

**Table 3:** The livelihood asset values of households in Sihe village in 1988, 1998, 2008, and 2016.

Asset Type	Year	Minimum	25th Percentile	Mean	Median	75th Percentile	Maximum
Natural assets	1988	0	0.016	0.035	0.027	0.041	0.932
	1998	0	0.021	0.046	0.035	0.052	0.913
	2008	0	0.039	0.084	0.052	0.078	0.901
	2016	0	0.023	0.046	0.033	0.049	0.967
Material assets	1988	0.003	0.017	0.039	0.025	0.032	0.973
	1998	0.009	0.081	0.138	0.109	0.143	0.893
	2008	0.104	0.258	0.388	0.322	0.536	0.836
	2016	0.276	0.539	0.606	0.616	0.687	0.902
Manpower assets	1988	0	0.296	0.368	0.357	0.447	0.973
	1998	0.055	0.224	0.295	0.284	0.362	0.706
	2008	0	0.308	0.389	0.389	0.459	0.758
	2016	0	0.332	0.412	0.407	0.493	0.769
Financial assets	1988	0	0.013	0.038	0.013	0.019	0.974
	1998	0	0.003	0.025	0.008	0.017	0.674
	2008	0	0.006	0.038	0.015	0.030	0.817
	2016	0	0.022	0.224	0.059	0.612	1

**Table 4:** The allocation of livelihood assets among households from 1988 to 2016.

Livelihood Assets	Interval	1988	1998	2008	2016
Natural assets	≤0.01	19	10	5	12
	0.01–0.02	40	34	8	28
	0.02–0.03	54	37	21	40
	0.03–0.04	25	34	31	35
	≥0.04	52	75	125	75
Material assets	≤0.4	190	182	112	13
	0.4–0.5	0	0	17	19
	0.5–0.6	0	0	38	51
	0.6–0.7	0	0	16	72
	≥0.7	0	8	7	35
Manpower assets	≤0.4	121	149	106	90
	0.4–0.5	41	30	51	54
	0.5–0.6	15	8	21	28
	0.6–0.7	10	2	9	11
	≥0.7	3	1	3	7
Financial assets	≤0.01	25	107	74	31
	0.01–0.02	119	44	50	16
	0.02–0.03	18	15	17	10
	0.03–0.04	9	7	25	10
	≥0.04	19	17	24	123

**Table 5:** Households relying on different number of livelihoods from 1988 to 2016.

Number of Livelihoods Adopted	1988		1998		2008		2016	
	Household Number	Proportion (%)	Household Number	Proportion (%)	Household Number	Proportion (%)	Household Number	Proportion (%)
1	40	21	12	6	6	3	7	4
2	108	57	74	39	41	22	36	19
3	39	21	86	45	103	54	97	51
4	3	2	17	9	37	19	44	23
5	0	0	1	1	3	2	6	3

**Table 6:** Livelihood diversity indexes of Sihe village and communities from 1988 to 2016.

Village/Community	1988	1998	2008	2016
Sihe village	2.01	2.57	2.93	3
Shangzhai	1.83	2.56	2.94	3.06
Nanjiayang	2	3	3	3.25
Dachawan	2.18	2.72	3.18	3.09
Napowan	1.93	2.53	2.93	3.07
Houwan	1.86	2.43	2.64	2.86
Fanwan	2.29	2.52	3.24	3.19
Leidian	1.88	2.62	2.92	3.19
Xiazhai	1.93	2.3	2.67	2.59
Liugeng	2.07	2.74	2.95	3.05

**Table 7:** Household classification from 1988 to 2016.

Household Type	Household Number				Standard for Classification
	1988	1998	2008	2016	
I	18	17	16	11	Natural asset value > 0.0525; the other asset values > corresponding average values.
II	44	58	81	33	Natural asset value: 0.035–0.0525; the other asset values < corresponding average values.
III	53	71	47	62	Natural asset value: 0.0175–0.035; the other asset values = corresponding average values.
IV	73	42	43	81	Natural asset value: 0–0.0175; the other asset values were high.
V	2	2	3	3	Natural asset value = 0

**Table 8:** Matrix showing changes in household livelihoods.

**Year**		**1998**					**In Total in 1988**
	**Household Type**	**I**	**II**	**III**	**IV**	**V**	
1988	**I**	6	11	1	0	0	18
	**II**	8	28	8	0	0	44
	**III**	3	9	38	2	1	53
	**IV**	0	10	24	39	0	73
	**V**	0	0	0	1	1	2
In total in 1998		17	58	71	42	2	190
**Year**		**2008**					**In Total in 1998**
	**Household Type**	**I**	**II**	**III**	**IV**	**V**	
1998	**I**	5	6	5	1	0	17
	**II**	5	36	17	0	0	58
	**III**	4	32	12	22	1	71
	**IV**	2	7	13	20	0	42
	**V**	0	0	0	0	2	2
In total in 2008		16	81	47	43	3	190
**Year**		**2016**					**In Total in 2008**
	**Household Type**	**I**	**II**	**III**	**IV**	**V**	
2008	**I**	2	6	3	5	0	16
	**II**	7	14	35	25	0	81
	**III**	2	13	8	24	0	47
	**IV**	0	0	16	27	0	43
	**V**	0	0	0	0	3	3
In total in 2016		11	33	62	81	3	190

Note: The number with the underscore is the number of households with no change in the type of livelihood in different periods.

**Table 9:** Indicators of land use scale in Sihe village and its communities from 1988 to 2016.

Land Use Indicators	Year	Sihe Village	Dachawan	Liugeng	Leidian	Houwan	Napowan	Fanwan	Nanjiayangpo	Shangzhai	Xiazhai
Total area of rural settlement plaques (hm^2^)	1988	8.75	0.95	1.92	0.88	0.42	0.61	1.11	0.45	1.26	1.16
	1998	9.65	1.1	2.1	1.05	0.51	0.66	1.11	0.47	1.29	1.34
	2008	10.23	1.31	2.25	1.09	0.58	0.72	1.11	0.47	1.29	1.39
	2016	10.23	1.31	2.25	1.09	0.58	0.72	1.11	0.47	1.29	1.39
Number of rural settlement plaques	1988	305	33	69	35	15	19	37	11	52	34
	1998	337	36	79	42	18	21	37	12	53	39
	2008	354	43	81	44	21	23	37	12	53	40
	2016	354	43	81	44	21	23	37	12	53	40
Average area of rural settlement plaques (m^2^)	1988	286.84	287.26	278.06	250.69	281.48	319.47	300.65	410.88	241.47	339.87
	1998	286.32	306.73	266.34	251.02	282.71	313.08	300.65	391.94	243.91	344.73
	2008	288.88	305.81	277.43	248.86	276.77	314.53	300.65	391.94	243.91	347.29
	2016	288.88	305.81	277.43	248.86	276.77	314.53	300.65	391.94	243.91	347.29

**Table 10:** The average rural settlement area per household for groups I–V in different periods (m^2^).

Year	I	II	III	IV	V
1988	302.76	296.24	288.40	285.37	224.55
1998	297.63	293.26	288.59	288.56	302.26
2008	296.80	289.56	289.21	290.84	303.97
2016	297.01	302.14	289.19	289.16	301.97
Mean	298.55	295.3	288.85	288.48	283.19

**Table 11:** The changes in the morphology of rural settlement plaques in Sihe village in 1988–2016.

Index	Year	Sihe Village	I	II	III	IV	V
Square index	1988	1.0192	1.0191	1.0139	1.0196	1.0198	1.0203
	1998	1.018	1.0190	1.0193	1.0190	1.0180	1.0192
	2008	1.0191	1.0195	1.0194	1.0192	1.0192	1.0205
	2016	1.0191	1.0191	1.0194	1.0191	1.0194	1.0196
Expansion index	1988	1.1505	1.1500	1.1440	1.1504	1.1507	1.1513
	1998	1.1498	1.1498	1.1501	1.1498	1.1486	1.1501
	2008	1.1500	1.1503	1.1503	1.1501	1.1500	1.1515
	2016	1.1500	1.1500	1.1503	1.1499	1.1502	1.1505
